# A Nanographene‐Porphyrin Hybrid for Near‐Infrared‐Ii Phototheranostics

**DOI:** 10.1002/advs.202309131

**Published:** 2024-03-02

**Authors:** Hao Zhao, Yu Wang, Qiang Chen, Ying Liu, Yijian Gao, Klaus Müllen, Shengliang Li, Akimitsu Narita

**Affiliations:** ^1^ Organic and Carbon Nanomaterials Unit Okinawa Institute of Science and Technology Graduate University 1919‐1 Tancha, Onna‐son, Kunigami‐gun Okinawa 904‐0495 Japan; ^2^ College of Pharmaceutical Sciences Soochow University Suzhou 215123 P. R. China; ^3^ Max Planck Institute for Polymer Research Ackermannweg 10 55128 Mainz Germany; ^4^ Department of Chemistry University of Oxford Chemistry Research Laboratory Oxford OX1 3TA UK; ^5^ Present address: Institute of Functional Nano & Soft Materials (FUNSOM) Soochow University Suzhou 215123 P.R. China

**Keywords:** nanographene, NIR‐II absorption, photoacoustic imaging, photothermal therapy, porphyrin

## Abstract

Photoacoustic imaging (PAI)‐guided photothermal therapy (PTT) in the second near‐infrared (NIR‐II, 1000–1700 nm) window has been attracting attention as a promising cancer theranostic platform. Here, it is reported that the π‐extended porphyrins fused with one or two nanographene units (NGP‐1 and NGP‐2) can serve as a new class of NIR‐responsive organic agents, displaying absorption extending to ≈1000 and ≈1400 nm in the NIR‐I and NIR‐II windows, respectively. NGP‐1 and NGP‐2 are dispersed in water through encapsulation into self‐assembled nanoparticles (NPs), achieving high photothermal conversion efficiency of 60% and 69%, respectively, under 808 and 1064 nm laser irradiation. Moreover, the NIR‐II‐active NGP‐2‐NPs demonstrated promising photoacoustic responses, along with high photostability and biocompatibility, enabling PAI and efficient NIR‐II PTT of cancer in vivo.

## Graphical abstract

Water‐dispersible nanoparticles containing two nanographene‐porphyrin hybrids (NGP‐1‐NPs and NGP‐2‐NPs) are fabricated, displaying intense absorption in NIR‐I and NIR‐II windows and achieving high photothermal conversion efficiency of 60% and 69%, respectively. NIR‐II‐active NGP‐2‐NPs demonstrate promising photoacoustic responses along with high photostability and biocompatibility, enabling the photoacoustic imaging and efficient NIR‐II photothermal therapy of cancer in vivo.

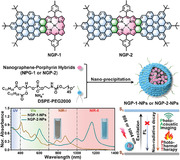

## 1 Introduction

Photoacoustic imaging (PAI) guided photothermal therapy (PTT), in which theranostic agents produce acoustic pressure waves and localized heat upon photoexcitation, has emerged as a promising theranostic platform for cancer treatments with high therapeutic efficiency and low toxicity.^[^ ^1^, ^2^, ^3^, ^4^ ^]^ In particular, theranostic agents with absorption in the second near‐infrared window (NIR‐II, 1000–1700 nm) have attracted increasing attention to achieve deeper tissue penetration while reducing the scattering of light, compared to those only active in the first NIR region (NIR‐I, 700–1000 nm).^[^ ^5^, ^6^, ^7^ ^]^ Various inorganic nanomaterials,^[^ ^8^, ^9^, ^10^ ^]^ such as nanocrystals of metals (i.e., Au and Fe)^[^ ^11^, ^12^, ^13^ ^]^ and 2D carbides/nitrides (MXenes),^[^ ^14^, ^15^ ^]^ and semiconducting conjugated polymers^[^ ^16^, ^17^ ^]^ have been explored as NIR‐II responsive PAI/PTT agents. However, their potential biodegradability, relatively low photothermal conversion efficiency (PCE), and/or structural inhomogeneity have precluded their further applications toward clinical trials.^[^ ^18^, ^19^ ^]^ To this end, NIR‐absorbing theranostic agents based on functional organic molecules have been extensively investigated in recent years, demonstrating better biocompatibility and efficient body clearance, enhancement of PCE based on the precise structural design, and thus promise for various applications in phototheranostics.^[^ ^20^, ^21^, ^22^, ^23^ ^]^ Different design strategies have been investigated to achieve bathochromic shifts of the optical spectra,^[^ ^24^, ^25^, ^26^, ^27^, ^28^ ^]^ including electron donor‐acceptor (D‐A) structures,^[^ ^20^, ^29^, ^30^, ^31^ ^]^ but the previous reports in the literature are mostly restricted to modulating absorption spectra in NIR‐I region, leaving it a challenge to develop NIR‐II‐absorbing molecular theranostic agents.^[^ ^19^, ^32^, ^33^ ^]^

Porphyrins, i.e., macrocyclic tetrapyrrole pigments, are promising theranostic agents for bioimaging and photodynamic/thermal therapies.^[^ ^34^, ^35^, ^36^, ^37^ ^]^ For example, natural porphyrin‐containing hemoglobin and nanothylakoids have enabled clinical PAI^[^ ^38^ ^]^ and multimodal cancer therapeutics,^[^ ^39^ ^]^ respectively, and a growing number of artificial porphyrinoid photosensitizers have been approved for clinal cancer photodynamic therapies.^[^ ^40^ ^]^ Despite these achievements, absorption of most of the porphyrin‐based theranostic agents is limited to visible light region (<700 nm).^[^ ^40^, ^41^, ^42^, ^43^, ^44^ ^]^ In the past decades, synthesis of π‐extended porphyrins fused with polycyclic aromatic hydrocarbons (PAHs) have been progressively reported, demonstrating NIR active optical properties,^[^ ^45^, ^46^, ^47^ ^]^ besides potential as nonlinear optical dyes.^[^ ^48^ ^]^ For instance, Anderson and co‐workers described the synthesis of multiple anthracene‐fused porphyrins,^[^ ^49^, ^50^, ^51^ ^]^ achieving markedly red‐shifted absorption spectra extending to the NIR‐II region. Wu et al. synthesized BODIPY‐fused porphyrins, which exhibited optical absorption at 1040 nm.^[^ ^52^ ^]^ On the other hand, Osuka and his colleagues reported NIR absorption of organic radicals based on porphyrin and its related structures,^[^ ^53^, ^54^ ^]^ and Furuta and co‐workers demonstrated generation of photoacoustic signals from a few bis‐metal complexes of expanded porphyrin analogues showing absorption up to NIR‐I or NIR‐II regions.^[^ ^55^, ^56^, ^57^ ^]^ However, all of these previous works did not include biological evaluations presumably due to the lack of water‐solubility, prohibiting further exploration of their bioapplications.

**Figure 1:**
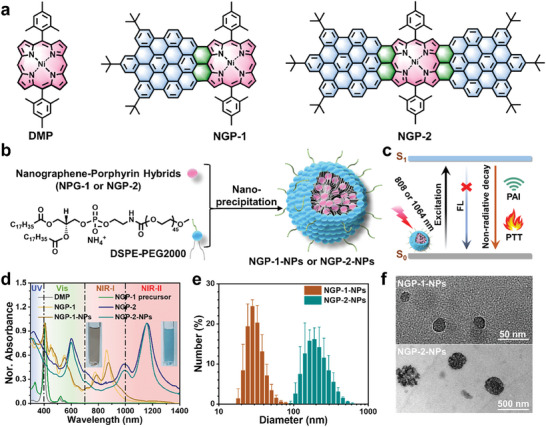
a) Chemical structures of DMP, NGP‐1, and NGP‐2. Schematic and conceptual illustration of b) the preparation of NGP‐NPs by a nanoprecipitation method and c) the PAI‐guided PTT based on NGP‐NPs. d) Normalized absorption spectra of DMP and NGP molecules in THF, as well as the NGP‐NPs in water. Inset: photographs of NGP‐1‐NPs (left) and NGP‐2‐NPs (right) solutions. e) Size distribution of NGP‐NPs by DLS experiments. f) TEM images of NGP‐NPs. Data shown in panel e are presented as mean ± standard deviation (n = 3).

Recently, we synthesized nanographene‐porphyrin hybrids (NGP‐1 and NGP‐2) by fusing one or two hexa‐*peri*‐hexabenzocoronene (HBC) with two K‐regions to a porphyrin core, namely 5,15‐(dimesityl)porphyrin nickel(II) (DMP), demonstrating the absorption in the NIR‐I (up to ≈1000 nm) and NIR‐II (up to ∼1400 nm) regions, respectively (**Figure** **1**a).^[^ ^58^ ^]^ In this work, we report the fabrication of water‐dispersible nanoparticles (NPs) containing NGP‐1 and NGP‐2, achieving high PCE of 60% and 69%, respectively, under 808 and 1064 nm laser irradiation. While NGP‐1‐NPs exhibited the robust NIR‐I active cancer cell killing performance, NGP‐2‐NPs revealed the promising photoacoustic responses and remarkable tumor elimination performance both in vitro and in vivo with high photostability and low toxicity, enabling PAI‐guided PTT in NIR‐II window. This work demonstrates the potential of such nanographene‐porphyrin hybrids for cancer phototheranostics and other bioapplications.

## 2 Results and Discussion

The syntheses and structural characterizations of NGP‐1 and NGP‐2 are described in our previous report.^[^ ^58^ ^]^ It is noted that the absorption of DMP and an oligophenylene‐based precursor of NGP‐1^[^ ^58^ ^]^ in tetrahydrofuran (THF) are in the visible light region with longest‐wavelength absorption peaks (*λ*_abs._) at 515 and 522 nm, respectively (Figure 1d; Figure S1, Supporting Information), precluding the NIR‐responsive photothermal conversation. In stark contrast, the π‐extended porphyrins fused with PAHs, like NGP‐1 and NGP‐2, demonstrate NIR absorptions, providing a powerful molecular engineering strategy toward NIR theranostics. For the preparation of water‐dispersible NPs, NGP‐1 and NGP‐2 molecules were reprecipitated with 1,2‐distearoyl‐sn‐glycero‐3‐phosphoethanolamine‐*N*‐[methoxy(polyethylene glycol)−2000] (DSPE‐PEG2000) through a nanoprecipitation method^[^ ^59^ ^]^ (Figure 1b). UV‐vis‐NIR absorption spectra indicated the intense longest‐wavelength absorption peaks (*λ*_abs._) of NGP‐1‐NPs and NGP‐2‐NPs locating at 873 and 1159 nm with the tail extending to 1000 and 1400 nm, respectively, marking their light‐harvesting capability and potential for photothermal conversion in NIR‐I and NIR‐II windows (Figure 1c,d). A slight red‐shift of 9 nm was observed for NGP‐1‐NPs in comparison to the *λ*_abs._ of NGP‐1 in tetrahydrofuran (THF), while that of NGP‐2‐NPs was virtually the same as NGP‐2 in THF, indicating the lack of significant aggregation‐induced effects. NGP‐1‐NPs and NGP‐2‐NPs in water were obtained as yellowish or blueish, and transparent dispersions without particles visible to the naked eyes, indicating the successful water‐dispersion of these π‐extended porphyrins (insets of Figure 1d). Dynamic light scattering (DLS) analysis of NGP‐1‐NPs and NGP‐2‐NPs revealed average diameters of 32 ± 3 and 215 ± 26 nm with polydispersity indices (PDI) of 0.49 and 0.14, respectively (Figure 1e), in agreement with the images obtained by transmission electron microscopy (TEM), which also revealed their uniform spherical shapes (Figure 1f). The larger size of NGP‐2‐NPs compared to NGP‐1‐NPs was presumably due to the extended planar structures of NGP‐2 molecules, while the dark spots in TEM images of these NGP‐NPs could be ascribed to high contrast of Ni(II) and benzene rings that are perpendicular to the substrate. No obvious changes in the size distributions of these NPs were observed after one week of storage in various solutions, including pure water, phosphate‐buffered saline (PBS), Dulbecco's modified Eagle medium (DMEM), and DMEM with 10% fetal bovine serum (FBS) (Figure S2, Supporting Information). In addition, the NGP‐1‐NPs and NGP‐2‐NPs in the same solutions showed negligible change in the UV‐vis‐NIR absorption spectra during five‐day storage at room temperature under ambient conditions (Figure S3 and S4, Supporting Information), highlighting their good stability in aqueous media.

**Figure 2:**
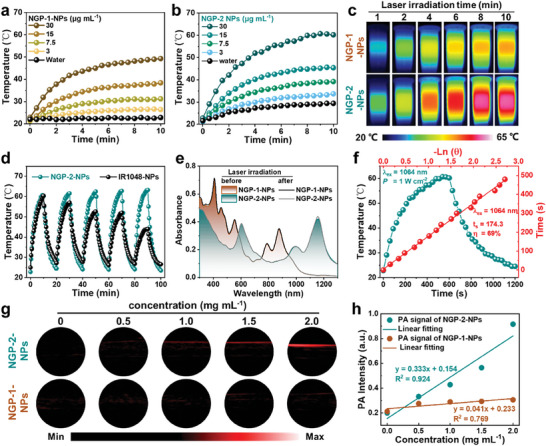
Photothermal curves of a) NGP‐1‐NPs under 808 nm laser irradiation (1 W cm^−2^), b) NGP‐2‐NPs under 1064 nm laser irradiation (1 W cm^−2^) for 10 min, and c) infrared images of NGP‐NPs with the extension of irradiation time. d) Photothermal stability of NGP‐2‐NPs and IR1048‐NPs under 1064 nm laser irradiation (1 W cm^−2^) for five on/off cycles. e) Absorption spectra of NGP‐1‐NPs and NGP‐2‐NPs before and after 808 nm (1 W cm^−2^) or 1064 nm (1 W cm^−2^) laser irradiation, respectively. The concentration of NGP‐1‐NPs and NGP‐2‐NPs was 30 µg mL^−1^, based on the amount of NGP‐1 or NGP‐2 molecule without DSPE‐PEG2000. f) Photothermal performance of NGP‐2‐NPs (30 µg mL^−1^, based on the amount of NGP‐2 without DSPE‐PEG2000) by cooling to room temperature with linear analysis. g) In vitro PA images of a glass capillary filled with NGP‐2‐NPs or NGP‐1‐NPs of different concentrations. h) PA signals of NGP‐2‐NPs or NGP‐1‐NPs showing a proportional relationship to concentration.

NIR‐enabling photothermal conversion of thus obtained two NGP‐NPs were evaluated with an infrared camera. Under 808 or 1064 nm (1 W cm^−2^) laser irradiation, the temperature of these two NGP‐NPs with various concentrations increased rapidly in a concentration‐dependent manner (**Figure** **2**a,b), which was clearly monitored by infrared images (Figure 2c; Figure S5, Supporting Information). After 10 min irradiation, NGP‐1‐NPs and NGP‐2‐NPs dispersions with an identical concentration of 30 µg mL^−1^ reached temperatures of 49.3 and 60.6 °C, respectively. Especially, the remarkable temperature increment of NGP‐2‐NPs triggered by 1064 nm laser indicated their capability of robust NIR‐II‐enabling photothermal conversion. The photostability of NGP‐2‐NPs was next assessed in comparison to NPs with a commonly used NIR‐II absorbing dye, IR1048 (IR1048‐NPs). While the highest temperatures reached by using IR1048‐NPs gradually decreased, from 60.2 to 44.1 °C after five laser on‐off cycles, NGP‐2‐NPs exhibited an undiminished photothermal conversion ability under the same condition, highlighting their excellent photostability in water (Figure 2d). Moreover, the absorption spectra of NGP‐1‐NPs and NGP‐2‐NPs displayed negligible changes before and after the laser irradiation, reinforcing their high photostability (Figure 2e; Figure S6a, Supporting Information). The PCE of NGP‐2‐NPs was calculated to be about 69% according to previously reported method (Figure 2f).^[^ ^60^ ^]^ Notably, the PCE of NGP‐2‐NPs is higher than the values of most of the previously reported NIR‐II absorbing photothermal agents, including organic small molecules,^[^ ^19^, ^32^, ^33^, ^61^, ^62^, ^63^ ^]^ supramolecular radicals,^[^ ^64^, ^65^ ^]^ semiconducting polymers,^[^ ^17^, ^66^, ^67^, ^68^, ^69^, ^70^, ^71^ ^]^ and inorganic materials^[^ ^11^, ^12^, ^13^, ^72^, ^73^ ^]^ (see Table S1 (Supporting Information) for the summary of PCEs of NIR‐II absorbing materials in the literature). On the other hand, NGP‐1‐NPs also exhibited promising PCE of 60% (Figure S6b, Supporting Information), which is among the highest values reported for NIR‐I absorbing agents in the literature (Table S2, Supporting Information). Negligible reactive oxygen species were detected during the irradiation process (Figure S7, Supporting Information), which is presumably due to the low‐lying excited states of the NGP molecules, prohibiting efficient energy transfer to oxygen molecules.^[^ ^57^, ^74^ ^]^ In addition, the heavy atom effect of the central Ni^2+^ ion^[^ ^58^, ^75^ ^]^ can also account for the favored non‐radiative relaxation to generate heat, contributing to the outstanding photothermal conversion performance of these NGP‐NPs. Moreover, NIR‐II light irradiation of NGP‐2‐NPs dispersions in a capillary tube also generated a PA signal (Figure 2g), which increased proportionally to their concentration (Figure 2h). In contrast, NGP‐1‐NPs dispersions resulted in negligible PA signal under identical conditions (Figure 2g,h), presumably due to their weak light‐harvesting capability in NIR‐II region. These results, demonstrating their high PCE, high photostability, and positive photoacoustic property, highlighted the potentials of NGP‐2‐NPs for PAI‐guided PTT in NIR‐II window.

**Figure 3:**
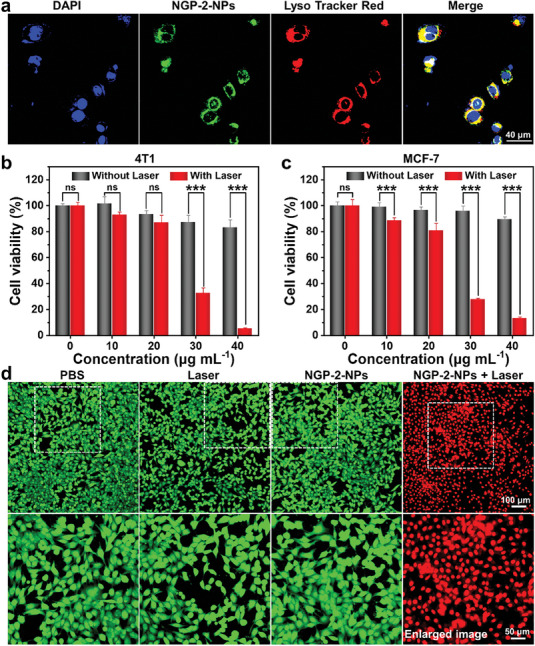
a) Co‐localization of NGP‐2‐NPs with DAPI and LysoTracker after incubating with 4T1 cancer cells for 4 h. Scale bar: 40 µm. Cell viability of b) 4T1 cells and c) MCF‐7 cells treated with various concentrations of NGP‐2‐NPs with or without 1064 nm laser irradiation (1 W cm^−2^) for 10 min. d) Live/dead images of 4T1 cells costained with AM (green fluorescence for live cells) and PI (red fluorescence for dead cells) after incubation with PBS or NGP‐2‐NPs with or without 1064 nm laser irradiation (1 W cm^−2^) for 10 min. Scale bar: 100 µm. The corresponding enlarged images for the white box region were also shown, Scale bar: 50 µm. Data shown in panels b and c are presented as mean ± standard deviation (*n* = 3). Probability (*P*)‐values are calculated by using one‐way ANOVA with Tukey test; ns: not significant, *p < 0.05, **p < 0.01, ***p < 0.001.

The intracellular distribution and cellular biocompatibility of NGP‐2‐NPs were subsequently evaluated by cell co‐localization analysis and cell viability assay, respectively, followed by the investigation of their performance for the in vitro NIR‐II laser activated photothermal ablation of cancer cell using the Live/Dead cell staining method (**Figure** **3**). Since the emission of NGP‐2 was quenched due to the heavy atom effect of the central Ni^2+^ ion, we used fluorescein‐labeled DSPE‐PEG2000 to fabricate NGP‐2‐NPs for confocal laser scanning microscopy (CLSM), aiming to reveal their intracellular distribution by cell co‐localization analysis with DAPI (cell nucleus dye) and LysoTracker Red. The green signal of fluorescein‐labeled NGP‐2‐NPs was located in the cell cytoplasm outside cell nucleus, and overlapped well with the signal of LysoTracker Red, resulting in a yellowish signal in the merged image and indicating the specific accumulation of fluorescein‐labeled NGP‐2‐NPs in lysosomes (Figure 3a). Moreover, fluorescein‐labeled DSPE‐PEG2000‐NPs without NGP‐2 molecules also demonstrated the accumulation in lysosomes (Figure S8, Supporting Information), eliminating the potential effect of fluorescein‐labeled DSPE‐PEG2000 on modulating the intracellular navigation of NGP‐2‐NPs. These NPs might follow the endocytic pathway from early endosomes to late endosomes and lysosomes, due to the absence of specific surface functionalization for organelle targeting.^[^ ^76^, ^77^ ^]^ The potential cytotoxicity of NGP‐2‐NPs was evaluated by a standard 3‐(4,5‐dimethylthiazol‐2‐yl)−2,5‐diphenyltetrazolium bromide (MTT) assay in three cell lines, including murine breast cancer cells (4T1), human breast cancer cells (MCF‐7), and normal mouse fibroblast cells (L929). Negligible cytotoxicity on the three cell lines was detected after incubation with NGP‐2‐NPs (0‐40 µg mL^−1^) for 24 h, displaying high cellular biocompatibility (Figure 3b,c; Figure S9, Supporting Information). In contrast, the cell viabilities of 4T1 and MCF‐7 cells remarkably decreased to ≈5% and 13%, respectively, when incubated with 40 µg mL^−1^ of NGP‐2‐NPs under 1064 nm laser (1 W cm^−2^) irradiation for 10 min (Figure 3b,c). According to the live/dead cell staining data, almost all 4T1 cells treated with NGP‐2‐NPs in the presence of 1064 nm laser irradiation were dead and stained by propidium iodide (PI), emitting red fluorescence. However, the cells in other control groups without NGP‐2‐NPs and/or the 1064 nm laser irradiation presented green fluorescence from calcein AM (AM), indicating their living state, which further validated the cellular biocompatibility of NGP‐2‐NPs in the dark and robust NIR‐II laser activated photothermal ablation of cancer cell (Figure 3d). Moreover, NGP‐1‐NPs also demonstrated the specific accumulation in lysosomes, low cellular toxicity, and robust NIR‐I laser triggered photothermal elimination of cancer cells (Figure S10, Supporting Information).

**Figure 4:**
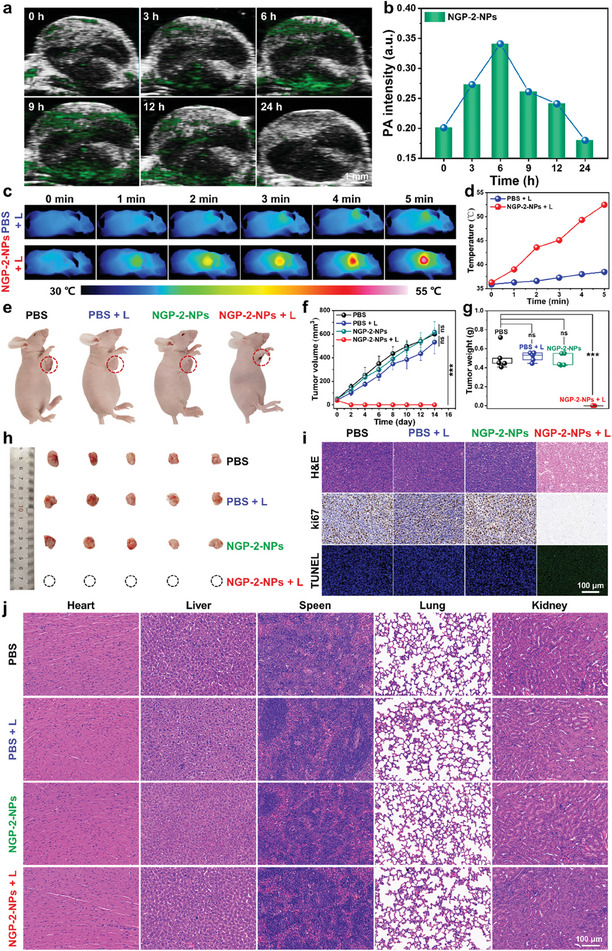
a) In vivo PA images of 4T1 tumor at different times post‐injection of NGP‐2‐NP through the tail vein. b) PA intensity from a) plotted as a function of time post‐injection. c) Infrared thermal images of tumor sites and d) corresponding temperature profiles of 4T1 tumor‐bearing mice with or without 1064 nm laser irradiation (1 W cm^−2^) for 10 min at 6 h post‐injection of PBS or NGP‐2‐NPs. e) Mice and h) tumor images at day 14 of different treatment groups. e) Tumor growth curves of different treatment groups for 14 days. f) Tumor volume and g) tumor weight of mice in different treatment groups during the therapy period. i) H&E, Ki67, and TUNEL staining of tumor tissue, as well as j) H&E staining of major organs excised from mice in different treatment groups. Scale bar: 100 µm. Data shown in panels f and g are presented as mean ± standard deviation (*n* = 5). *P*‐values are calculated by using one‐way ANOVA with Tukey test; ns: not significant, *p < 0.05, **p < 0.01, ***p < 0.001.

NIR‐II activated PAI and photothermal ablation in vivo using NGP‐2‐NPs were next explored on 4T1 tumor‐bearing mice. To perform the in vivo PAI with the aim of visualizing solid tumors, tumor‐xenografted mice were intravenously injected with NGP‐2‐NPs in PBS buffer (100 µL, 1 mg mL^−1^), and the PA images were recorded at different time intervals via the PA computed tomography system. The PAI results revealed that NGP‐2‐NPs started to illustrate the tumor margin at 3 h post‐injection, reached the highest passive accumulation at approximately 6 h post‐injection through blood circulation, and were then almost completely excreted after 24 h post‐injection (**Figure** **4**a,b), suggesting the considerable tumor‐penetration and tumor‐targeting ability of NGP‐2‐NPs. On the other hand, a time frame of 6 h post‐injection was selected for photothermal treatment, considering the highest accumulation of NGP‐2‐NPs in the tumor sites.

To evaluate the in vivo photothermal therapeutic efficacy, 4T1 tumor‐bearing mice were randomly classified to four groups, namely PBS, laser, PBS + laser, and NGP‐2‐NPs + laser. NGP‐2‐NPs in PBS buffer (100 µL, 1 mg mL^−1^) were intravenously injected on mice followed by 1064 nm laser irradiation (1 W cm^−2^) at 6 h post‐injection, while the temperature increment on tumor sites as well as inhibition of tumor growth was monitored. The mice in NGP‐2‐NPs + laser group exhibited a rapid increment of tumor temperature to ≈52.5 °C after 5 min of laser irradiation, which was already beyond the requirement for inducing tumor hyperthermia (45 °C). In contrast, the tumor temperature in the control group of PBS + laser showed a mild rise only up to 38.5 °C at the end of irradiation. An infrared camera clearly recorded the treatment process, revealing the robust in situ photothermal conversion of NGP‐2‐NPs enabled by NIR‐II light (Figure 4c,d). Notably, the tumors displayed consistently high growth rates in the three control groups. In stark contrast, the treatment of NGP‐2‐NPs under laser irradiation completely eliminated the tumors without any recurrence in the subsequent 14 days, demonstrating the promising photothermal therapeutic performance enabled by NGP‐2‐NPs (Figure 4e–h). The apoptosis and bioactivity of residual tumor tissues was further examined via the methods of tumor biopsy, such as hematoxylin and eosin (H&E) staining, immunohistochemistry analysis with Ki67 marker (proliferating cell marker) and terminal deoxynucleotidyl transferase dUTP nick end labeling (TUNEL) assay (Figure 4i). Efficient apoptosis of malignant cells was achieved in the group of NGP‐2‐NPs + laser, as evidenced by the highly fragmentized nucleus, the absence of Ki67 marker, and the intense TUNEL signal, while other treatments had negligible influence on the tumor growth. These results revealed that NGP‐2‐NPs can passively accumulate in the tumor region and effectively activate tumor apoptosis through NIR‐II responsive PTT in vivo.

The in vivo biocompatibility of NIR‐II enabling PTT using NGP‐2‐NPs was systematically examined by mice body weight monitoring, histological staining, and blood analysis. Unobvious variations in the body weight steadily indicated negligible adverse effects of NGP‐2‐NPs and laser irradiation to the mice during the treatment period (Figure S11, Supporting Information). In addition, the H&E staining results revealed no detectable damage in the organs, including heart, liver, spleen, lungs, and kidneys isolated from treated tumor‐bearing mice in the four groups (Figure 4j). The results of complete blood panel analysis (i.e., red blood cell numbers (RBC), mean corpuscular volume (MCV), red blood cell distribution width (RDW), mean corpuscular hemoglobin (MCH), mean corpuscular hemoglobin concentration (MCHC), platelet numbers (PLT), mean platelet volume (MPV), and lymphocyte numbers (Lymph)) demonstrated no noticeable infection and inflammation in the mice body (Figure S12, Supporting Information). Moreover, the analysis of blood biochemistry (i.e., aspartate transaminase (AST), alanine transaminase (ALT), creatinine (CREA), and urea) confirmed rare side effect on the liver and kidney functions after treated with NGP‐2‐NPs upon irradiation (Figure S13). The above findings clearly revealed a high biocompatibility of NGP‐2‐NPs without biotoxicity in vivo.

## 3 Conclusion

In summary, we achieved dispersion of nanographene‐porphyrin hybrids in various aqueous media through encapsulation into amphiphilic polymer nanoparticles (NGP‐1‐NPs and NPG‐2‐NPs), showing intense absorption extending to ≈1000 and ≈1400 nm in the NIR‐I and NIR‐II windows, respectively. NGP‐1‐NPs and NGP‐2‐NPs demonstrated high PCEs of 60% and 69%, respectively, with remarkable photostability, biocompatibility, and high therapeutic efficacy both in vitro and in vivo. Moreover, NGP‐2‐NPs exhibited promising photoacoustic responses, which enabled the visualization of the dynamic processes of their accumulation in tumors. The PTT could thus be guided by the PAI through the NIR‐II window, for the first time using a porphyrin‐based dye. This strategy can potentially be applied to achieve water‐dispersion of other expanded porphyrins, possibly even porphyrin tapes^[^ ^78^ ^]^ and porphyrin‐incorporated graphene nanoribbons,^[^ ^79^ ^]^ providing new materials for NIR‐II nanomedicine. Moreover, such NPs can be surface‐functionalized through known methods to realize the targeting of cancer cells^[^ ^17^ ^]^ or neural ion channels,^[^ ^80^ ^]^ expanding their potential to NIR‐II‐based nanotheranostics and optogenetics.

## Conflict of Interest

The authors declare no conflict of interest.

## Supporting information

Supporting Information

## Data Availability

The data that support the findings of this study are available from the corresponding author upon reasonable request.
