# Supplementary material for: A Nanographene‐Porphyrin Hybrid for Near‐Infrared‐Ii Phototheranostics
Source: Adv Sci (Weinh). 2024 Mar 2;11(18):2309131. doi: 10.1002/advs.202309131 (PMC11095198; doi:10.1002/advs.202309131)
Supplement: Supplementary file 1 — Supporting Information [file ADVS-11-2309131-s001.pdf]

ADVANCED SCIENCE
Open Access

Supporting Information

for *Adv. Sci.*, DOI 10.1002/advs.202309131

A Nanographene-Porphyrin Hybrid for Near-Infrared-Ii Phototheranostics

Hao Zhao, Yu Wang, Qiang Chen, Ying Liu, Yijian Gao, Klaus Müllen, Shengliang Li and Akimitsu Narita**

Supporting Information

A Nanographene-Porphyrin Hybrid for Near-Infrared-II Phototheranostics

Hao Zhao[#], Yu Wang[#], Qiang Chen, Ying Liu, Yijian Gao, Klaus Müllen, Shengliang Li[*], and Akimitsu Narita[*]

H. Zhao, A. Narita
Organic and Carbon Nanomaterials Unit, Okinawa Institute of Science and Technology Graduate University, 1919-1 Tancha, Onna-son, Kunigami-gun, Okinawa 904-0495, Japan

Y. Wang, Y. Liu, Y. Gao, S. Li
College of Pharmaceutical Sciences, Soochow University, Suzhou, 215123, P.R. China
E-mail: lishengliang@suda.edu.cn

Q. Chen, K. Müllen, A. Narita
Max Planck Institute for Polymer Research, Ackermannweg 10, 55128 Mainz, Germany
E-mail: narita@mpip-mainz.mpg.de

Q. Chen
Department of Chemistry, University of Oxford, Chemistry Research Laboratory, Oxford OX1 3TA, United Kingdom
Current address: Institute of Functional Nano & Soft Materials (FUNSOM), Soochow University, Suzhou, 215123, P.R. China

[#]These authors contributed equally to this work.

Experimental Section

Materials. All the chemicals were purchased from commercial suppliers and used as received unless otherwise noted. Nanographene-fused porphyrins (NGP-1 and NGP-2) were prepared as we previously described.[1] 1,2-Distearoyl-sn-glycero-3-phosphoethanolamine-*N*-[methoxy(polyethylene glycol)-2000] (DSPE-PEG2000) was purchased from Yarebio technology Co., Ltd (Shanghai, China). Fluorescein-labeled DSPE-PEG2000 was obtained from Ponsure Biotechnology company (Shanghai, China). 2,7-Dichlorofluorescin diacetate (DCFH-DA) and IR-1048 were purchased from Sigma-Aldrich (Shanghai, China). Mice breast cancer cells (4T1) and human breast cancer cells (MCF-7) were provided by the Institute of Basic Medical Sciences, Chinese Academy of Medical Sciences (Beijing, China). Normal mouse fibroblast cells (L929) were obtained from Procell Life Science&Technology Co., Ltd (Wuhan, China). Dulbecco's Modified Eagle Medium (DMEM), penicillin-streptomycin and fetal bovine serum (FBS) was purchased from ThermoFisher Scientific Co., Ltd (Shanghai, China). Phosphate buffer saline (PBS) was purchased from Servicebio Technology Co., Ltd (Wuhan, China). Pancreatin was purchased from New Cell & Molecular Biotech Co., Ltd (Suzhou, China). 3-(4,5-Dimethylthiazol-2-yl)-2,5-diphenyltetrazolium bromide (MTT) was commercially obtained from Keqing Biotechnology Co., Ltd (Suzhou, China). 4’,6-Diamidino-2-phenylindole (DAPI), Calcein-AM and propidium iodide (PI) were provided by Beijing Solarbio Science & Technology Co., Ltd (Beijing, China). LysoTracker™ Deep Red was purchased from ThermoFisher Scientific Co., Ltd (Shanghai, China). Deionized (DI) water was obtained from a Merck Millipore Direct-Q 5 UV system (Shanghai, China).

Measurements. Absorption spectra were recorded on a UV-vis-NIR spectrophotometer (PE950, Perkin Elmer, USA). Laser machines (808 nm and 1064 nm) were purchased from Changchun New Industries Optoelectronics Tech. Co., Ltd (Changchun, China). The temperature variations were recorded with a thermal imaging camera (Fluke Ti400, IR Fusion

Technology, USA). Photoacoustic (PA) images were taken by an ultra-high resolution multi-mode ultrasonic photoacoustic imaging system (Vevo LAZR, Visual Sonics, Canada). Confocal laser scanning microscopy (CLSM) images were taken on a confocal laser scanning microscope (Nikon A1R HD25, Japan). The MTT assay was performed on a microplate reader (Tecan Infinite M1000 PRO, Switzerland).

Preparation of NGP-1-NPs and NGP-2-NPs. Water dispersible nanoparticles of NGP-1 and NGP-2 (NGP-1-NPs and NGP-2-NPs, respectively) were prepared through a standard nanoprecipitation method.[2] 0.5 mg of NGP-1 and 5 mg of DSPE-PEG2000 were dissolved in 1 mL of tetrahydrofuran (THF) to obtain a homogeneous solution, which was quickly added into 9 mL of DI water under vigorously stirring. The resulting mixture was stirred at room temperature with continuous nitrogen bubbling for 48 h to completely remove THF. The obtained dispersion of NGP-1-NPs was concentrated to 1 mL by centrifugal filter units (100 kDa, Millipore) at 2000 rpm (revolutions per minute) for 5 min, and then diluted with DI water to 9 mL, followed by concentration to 1 mL again by the centrifugation. This procedure was repeated for three times in total. This concentrated dispersion of NGP-1-NPs was stored in the dark at 4 °C. The concentrated NGP-2-NPs solution was prepared by the same procedure. Fluorescein-labeled NGP-1-NPs and NGP-2-NPs was also prepared by the same method, using 10 wt.% of fluorescein-labeled DSPE-PEG2000, namely 0.5 mg of fluorescein-labeled DSPE-PEG2000 and 4.5 mg of DSPE-PEG2000 as the amphiphilic polymer.

Dynamic Light Scattering (DLS) Analysis of NGP-1-NPs and NGP-2-NPs. DLS measurements were performed using a Zetasizer Nano ZS 90 instrument (Malvern, UK) equipped with a He–Ne laser (633 nm, 4 mW). Measurements were carried out in a polystyrene cuvette at a 173° accumulation angle after equilibrating for 2 min at 25 °C. The data were processed by the instrument software (Zetasizer Nano software v3.30) to give the number mean particle size and polydispersity index value by non-negative least squares method.

Transmission Electron Microscopy (TEM) Analysis of NGP-1-NPs and NGP-2-NPs. TEM images were taken on a transmission electron microscope (FEI Tecnai F20, USA) with the acceleration voltage of 200.0 kV. 10 μL of a dispersion of NGP-1-NPs or NGP-2-NPs with a concentration of 10 μg mL^{-1} (based on the amount of NGP-1 or -2) was deposited on a microgrid precoated with ultra-thin carbon film (230 mesh, Beijing Zhongjingkeyi Technology Co., Ltd, Beijing, China) and dried under air overnight. The sample was further dried with an infrared baking lamp (LP23030-B, Beijing Zhongjingkeyi Technology Co., Ltd, Beijing, China) for 10 min prior to the TEM observations.

Photothermal Conversion and Photothermal Stability of NGP-1-NPs and NGP-2-NPs. Dispersions (0.5 mL) of NGP-1-NPs with various concentrations (30, 15, 7.5, and 3.0 μg mL^{-1}) in DI water were irradiated with 808 nm laser (1 W cm^{-2}) for 10 min to reach the maximum temperature plateau, and then the laser was turned off for natural cooling. Similarly, dispersions (0.5 mL) of NGP-2-NPs with various concentrations (30, 15, 7.5, and 3.0 μg mL^{-1}) in DI water were irradiated with 1064 nm laser (1 W cm^{-2}) for 10 min, followed by natural cooling. Temperature variations during these processes were recorded with the Ti400 thermal imaging camera. DI water was applied as control under the same experiment conditions. The photothermal stability of NGP-1-NPs and NGP-2-NPs was determined through laser irradiation for 5 cycles (laser on/off), and that of NGP-2-NPs (30 μg mL^{-1}) and IR-1048-NPs (100 μg mL^{-1}) was compared under 1064 nm laser (1 W cm^{-2}).

Photothermal Conversion Efficiency Calculations. The photothermal conversion efficiencies (η) were determined according to the procedure described by Roper[3] and Li.[4] 0.5 mL of NGP-2-NPs dispersion (30 μg mL^{-1}) in DI water was irradiated with 1064 nm laser (1 W cm^{-2}) for 10 min to reach a maximum equilibrium temperature (T_{max}). Then, the laser was turned off and the sample was allowed to naturally cool to a temperature in equilibrium with its surroundings (T_{surr}). The temperature variations of NGP-2-NPs dispersion during the natural

cooling period were recorded with the Ti400 thermal imaging camera. Based on the obtained photothermal curves, η was calculated using the following equation:

$$\eta = \frac{hS\Delta T_{max} - Q_{Dis}}{I(1 - 10^{-A_{1064}})}$$

where ΔT_{max} is the maximum temperature change that is calculated according to T_{max}–T_{surr}, Q_{Dis} is the heat related to the laser absorbance of DI water and container, I is laser power density (1 W cm^{-2} in this work), A_{1064} is the absorbance value of the sample at 1064 nm, h is the heat transfer coefficient, S is the surface area of the container, and the value of hS (W °C^{-1}) was calculated with the following equation:

$$\tau_S = \frac{m_D C_D}{hS}$$

where m_D is the mass (0.5 g), C_D is the heat capacity (4.2 J g^{-1} °C^{-1}) of DI water used as the solvent, and τ_s is the sample system time constant, which was calculated with the following equation:

$$\mathrm{t} = -\tau_S(\ln\theta)$$

where t is time starting from turning off the laser (0 s) during the cooling period, ΔT is the temperature change that is calculated according to T_t–T_{surr} (T_t is the temperature of the system at the time of t), θ is the ratio of ΔT to ΔT_{max} during the cooling period. From the slope of the red line displayed in **Figure 2f** of the main text, τ_s was calculated to be 174.3 s. The η of NGP-1-NPs was determined in the same way using 808 nm laser (1 W cm^{-2}).

Reactive Oxygen Species (ROSs) Production by NGP-1-NPs and NGP-2-NPs. The production of ROSs by NGP-1-NPs and NGP-2-NPs under laser irradiation was detected with 2′,7′-dichlorofluorescin (DCFH) as the probe.[2] A dispersion of NGP-1-NPs (30 μg mL^{-1}) or NGP-2-NPs (30 μg mL^{-1}) and DCFH (40 μM) in 2 mL of DI water was irradiated with 808 nm laser (1 W cm^{-2}) or 1064 nm laser (1 W cm^{-2}), respectively. The fluorescence of the dispersion at 525 nm was collected every minute with an excitation wavelength of 460 nm. As control

experiments, aqueous solutions of DCFH (40 μM) only were also analyzed under the same laser irradiation conditions.

Cell Culture. L929, 4T1, and MCF-7 cells were cultured in DMEM supplemented with 10% FBS, 1% penicillin, and streptomycin in a humidified incubator containing 5% CO_2 at 37 °C.

***In Vitro* Photo-Induced Cytotoxicity of NGP-1-NPs and NGP-2-NPs.** The cytotoxicity of NGP-1-NPs and NGP-2-NPs to 4T1 or MCF-7 cancer cells was evaluated by the MTT assay. 4T1 or MCF-7 cancer cells were seeded in 96-well plates at a density of 4×10^3 cells/well and cultured in fresh cultured medium at 37 ℃ overnight to achieve the 60-70% confluence. For the dark groups, the above cells were further incubated with various concentrations of NGP-1-NPs or NGP-2-NPs (40, 30, 20, 10, and 0 μg mL^{-1}) in fresh cell culture medium for another 24 h incubation at 37 ℃. Then, MTT (0.5 mg mL^{-1} in DMEM without FBS, 100 μL/well) was added with another 4 h incubation at 37 °C. After that, 100 μL of dimethyl sulfoxide (DMSO) was employed to thoroughly dissolved the produced formazan. After shaking the plates for 1 min, the absorbance of all of the wells at 490 nm was read with a microplate reader. The cell viability rate (VR) was calculated according to the following equation:

$$VR = A/A_0 \times 100\%$$

where A is the absorbance of the experimental group and A_0 is the absorbance of the control group (0 μg mL^{-1}). For the laser irradiation groups, when replacing with fresh medium mixed with various concentrations of NPs for another 4 h incubation at 37 ℃, the cells were illustrated with 808 nm laser (1 W cm^{-2}) or 1064 nm laser (1 W cm^{-2}) for 10 min. After another 24 h incubation, MTT and DMSO were subsequently added as described above. Moreover, the dark cytotoxicity of NGP-2-NPs to normal L929 cells was also analyzed using MTT assay.

Cell Co-localization Analysis of NGP-1-NPs and NGP-2-NPs. The cell co-localization of NGP-1-NPs and NGP-2-NPs was analyzed by CLSM imaging. 4T1 cells were seeded in

confocal dishes at a density of 4×10^3 cells/well and cultured in the medium at 37 °C overnight. The cells were incubated with fluorescein-labeled NGP-1-NPs (40 μg mL^{-1}) or fluorescein-labeled NGP-2-NPs (40 μg mL^{-1}) in fresh cell culture medium for 4 h. Then, the cells were co-stained with DPAI (1 μM)/LysoTracker Red (1 μM) for another 30 min. The cells were washed with PBS for three times followed by the CLSM imaging. The CLSM images of DAPI, FITC-labeled NGP-1/NGP-2-NPs, and LysoTracker, were collected at the ranges of 420-460 nm (λ_{ex}: 405 nm), 500-600 nm (λ_{ex}: 488 nm), and 660-700 nm (λ_{ex}: 647 nm), respectively.

Live/dead Cell Imaging. Live/dead cell staining assay was performed by confocal laser scanning microscopy. 4T1 cancer cells were seeded in confocal dishes at a density of 8×10^4 cells/well and cultured in the medium at 37 °C for 12 h. The cells were incubated with NGP-1-NPs (40 μg mL^{-1}) in fresh cell culture medium for 4 h, which were illuminated with 808 nm laser (1 W cm^{-2}) for 10 min followed by another 4 h incubation. After removing the medium, the cells were incubated with fresh medium containing Calcein-AM (2 μM) and PI (4.5 μM) for 30 min at 37 °C. CLSM images were acquired after washing with PBS for three times. The CLSM images of Calcein-AM and PI were collected at 500-540 nm (λ_{ex}: 488 nm) and 570-650 nm (λ_{ex}: 561 nm), respectively.

***In Vivo* Mouse Tumor Model.** The protocols of the animal experiments are approved by the Institutional Ethical Committee of Animal Experimentation of Soochow University in China (No. 202337031), and the experiments are conducted according to governmental and international guidelines on animal experimentation. All BALB/C nude mice (female, 6-8 weeks) with body weights of 18-20 g were purchased from Chang Zhou Cavens Laboratory Animal Ltd (Changzhou, China). 4T1 cells (100 μL, 2×10^7 cell mL^{-1}) was injected subcutaneously into the right upper extremity subcutaneously to establish a xenografted tumor model. The *in vivo* PA imaging, and *in vivo* evaluation of PTT efficacy were performed until the tumor size reached ~100 mm^3. The length (A) and wide (B) of the tumor were measured by

a vernier caliper. The tumor volume (V_{tumor}) was calculated according to the following equation:

$$V_{tumor} = (A \times B^2)/2$$

Photoacoustic (PA) Imaging Using NGP-2-NPs. The PA imaging performances of NGP-2-NPs *in vitro* and *in vivo* were evaluated by Vevo LAZR, respectively. For *in vitro* PA imaging, the dispersions of NGP-2-NPs with various concentrations (2.0, 1.5, 1.0, and 0.5 mg mL^{-1}) in PBS was used to examine the photoacoustic signals and their linear relation. For *in vivo* PA imaging, the 4T1 tumor-bearing mice were intravenously injected with 100 μL of NGP-2-NPs dispersion (1 mg mL^{-1}) according to requirement of 10 mg kg^{-1}, and then the PA signal of the tumor was collected at various post-injection times (0, 3, 6, 9, 12, and 24 h).

***In Vivo* PTT of NGP-2-NPs.** 4T1 tumor-bearing mice were randomly divided into 4 groups (n = 5): PBS group, NGP-2-NPs group, PBS + Laser group, and NGP-2-NPs + Laser group. Experimental mice were intravenously injected with PBS (100 μL) or NGP-2-NPs aqueous dispersion (100 μL, 1 mg mL^{-1}) in PBS at 6 h post-injection, and the tumor sites were irradiated with 1064 nm laser (1 W cm^{-2}) for 10 min. During the irradiation process, temperature changes of the mice in the NGP-2-NPs + Laser group were recorded with the Ti400 thermal imaging camera. The body weight and tumor volume of the mice in every group were monitored every 2 days during the following 14 days (the day of the laser treatment was set as day 0). At the end of the therapeutic process, all of the mice were sacrificed and the tumors were harvested for evaluating the antitumor effect.

Histomorphology analysis. At 12 h post-treatment, the tumor from the various group was collected and immediately fixed in a 4% formaldehyde solution. Then, the tumor tissue was sectioned into 10 μm thickness by histotome for further hematoxylin and eosin (H&E) staining, as well as Ki67 and fluorescent terminal deoxynucleotidyl transferase dUTP nick end labeling (TUNEL) staining. To further evaluate the biosecurity, the major organs mainly including heart,

liver, spleen, lung, and kidney were harvested after the treatment process for further H&E staining.

Biochemical Analysis. To evaluate the biocompatibility, the blood samples were collected at the end of treatments to test the main hematology markers and blood biochemical parameters. Blood and serum are collected for blood biochemistry tests and complete blood panel analysis by an automated spectrophotometric analyzer ($n = 3$). Data are presented as mean ± standard deviation ($n = 3$ independent samples). The blood sample from the untreated mice was used as a control group to compare the biosecurity.

Statistical Analysis. The data are plotted by Origin software 2021. The sample sizes in statistical analysis are $n = 3$ or 5 unless otherwise noted, while the data are shown as mean ± standard deviation. Probability (*P)*-values are calculated by using one-way ANOVA with Tukey test, *$P < 0.05$, **$P < 0.01$, ***$P < 0.001$.

Supplementary Data

Table S1. Summary of NIR-II absorbing materials

Materials	Abs. (nm)	Operating laser (nm)	PCE (%)	Bioapplications	Ref.
**Organic small molecules**					
BAF4 NPs	1000	1064	80	PAI-guided NIR-II PTT of cancer	*Angew. Chem. Int. Ed.* **2021**, 60, 22376-22384.
IR-SS NPs	1120	1064	77	PAI-guided NIR-II PTT of cancer	*Adv. Mater.* **2020**, 32, 2001146.
SW8@NPs	broad	1064	75	NIR-II PTT of osteosarcoma	*Research* **2023**, 6, 0169.
**NGP-2-NPs**	**1159**	**1064**	**69**	**PAI-guided NIR-II PTT of cancer**	**This work**
NA1020@PLX	1020	1064	61	NIR-II PTT of osteosarcoma	*Adv. Mater.* **2023**, 35, 2301901.
N1@2P NPs	broad	1064	53.8	PAI-guided NIR-II PTT of cancer	*Small* **2023**, 19, 2300203.
Nano-BFF	broad	1064	34.3	PAI-guided NIR-II PTT of cancer	*Nat. Commun.* **2021**, 12, 218.
**Supramolecular radicals**					
$(NDI\text{-}2CB[7])^{\bullet -}$	broad	1064	66.9	NIR-II PTT of bacterial	*Angew. Chem. Int. Ed.* **2023**, 62, e20230851.
$2MPT^{\bullet +}\text{-}CB[8]$	1004	1064	54.6	NIR-II PTT of cancer	*Angew. Chem. Int. Ed.* **2019**, 58, 15526-15531.
**Semiconducting polymers**					
Polypyrrole (PPy) nanosheets	broad	1064	64.6	NIR-II PTT of cancer	*Nano Lett.* **2018**, 18, 2217-2225.
DPP-BTzTD NPs	broad	1064	53	PAI-guided NIR-II PTT of cancer	*Adv. Funct. Mater.* **2020**, 30, 1909673.
TBDOPV-DT NPs	broad	1064	50	PAI-guided NIR-II PTT of cancer	*ACS Appl. Mater. Interfaces* **2018**, 10, 7919-7926.
SP4 NPs	1083	1064	46.5	PAI-guided NIR-II PTT of cancer	*Angew. Chem. Int. Ed.* **2023**, 62, e202301617.

SPN_{I-II}	1059	1064	43.4	Fluorescence-imaging-guided NIR-II PTT of cancer	*Adv. Mater.* **2018**, 30, 1705980.
PSQPNs-DBCO	broad	1064	33.4	Fluorescence-imaging-guided NIR-II PTT of cancer	*Biomaterials* **2020**, 243, 119934
P1 NPs	broad, near 1064	1064	30.1	PAI-guided NIR-II PTT of cancer	*Adv. Mater.* **2018**, 30, 1802591.
**Inorganic material**					
2D niobium carbides	broad	1064	45.6	NIR-II PTT of cancer	*J. Am. Chem. Soc.* **2017**, 139, 16235-16247.
$Au@Cu_{2-x}S$ nanocrystals	broad	1064	43	NIR-II PTT of cancer	*Adv. Mater.* **2016**, 28, 3094-3101.
Cu_3BiS_3 nanorods	broad	1064	40.7	PAI-guided NIR-II PTT of cancer	*Biomaterials* **2017**, 112 164-175.
$Au\text{-}Cu_9S_5$	broad	1064	37	X-ray CT imaging-guided NIR-II PTT of cancer	*J. Am. Chem. Soc.* **2014**, 136, 15684-15693.
$Fe_3O_4@CuS$	broad	1064	19.2	Magnetic resonance imaging (MRI)-guided NIR-II PTT of cancer	*Adv. Funct. Mater.* **2015**, 25, 6527-6537.

Table S2. Summary of NIR-I absorbing materials

Materials	Abs. (nm)	Operating laser (nm)	PCE (%)	Bioapplications	Ref.
**Organic small molecules**					
tfm-BDP NPs	810	808	88.3	PAI-guided NIR-I PTT of cancer	*Adv. Mater.* **2020**, 32, 1907855
QDI-NPs	~710	808	64.7	PAI-guided NIR-I PTT of cancer	*Angew. Chem. Int. Ed.* **2019**, 58, 1638-1642.
**NGP-1-NPs**	**873**	**808**	**60**	**NIR-I PTT of cancer**	**This work**
2TPE-NDTA-doped NPs	broad	808	54.9	PAI-guided NIR-I PTT of cancer	*Nat. Commun.* **2019**, 10, 768

BT6 NPs	~660	808	36	NIR-II fluorescence imaging-guided NIR-I PTT of cancer	*Adv. Mater.* **2023,** 35, 2211632.
FDA-approved ICG	777	808	15.1	NIR-I PTT of cancer	*ACS Appl. Mater. Interfaces* **2014**, 6, 6709-6716.
**Supramolecular radicals**					
PDI radical anions	818	808	35.7	NIR-I PTT of cancer	*J. Am. Chem. Soc.* **2022**, 144, 2360-2367.
**Semiconducting polymers**					
Pdots-1	816	808	65	NIR-I PTT of cancer	*Chem. Mater.* **2016**, 28, 8669-8675.
DSP	635	808	44.2	NIR-I photothermal for remote activation of gene expression	*Angew. Chem. Int. Ed.* **2017**, 56, 9155-9159.
SPFeN/SPCN	620/660	808	42/38	NIR-I photothermal ferrotherapy of Cancer	*Angew. Chem. Int. Ed.* **2020**, 59, 10633-10638.
PDPP-DBT NPs	broad	808	34	NIR-I photothermal for remote control of gene expression	*Adv. Mater.* **2018,** 30, 1705418.
CPNs	broad	808	26.9	Activation of cancer immunotherapy using NIR-I PTT	*Adv. Mater.* **2021**, 33, 2102570.
**Inorganic material**					
V_2C nanosheet	broad	808	48	NIR-I PTT of cancer	*Angew. Chem. Int. Ed.* **2020**, 59, 6601-6606.
$WO_{2.9}$ nanorods	broad	808	44.9	NIR-I PTT of cancer	*Angew. Chem. Int. Ed.* **2018**, 57,10666-10671.
GDY-PEG	broad	808	42	NIR-I PTT of cancer	*Chem. Mater.* **2017**, 29, 6087-6094.
MoS_2 nanosheets	broad	808	24.3	NIR-I photothermal-triggered drug delivery for cancer therapy	*ACS Nano* **2014**, 8, 7, 6922-6933.
Au nanorods	broad	broad	17	NIR-I photothermal evaluation	*J. Am. Chem. Soc.* **2016**, *138, 9049-9052.*

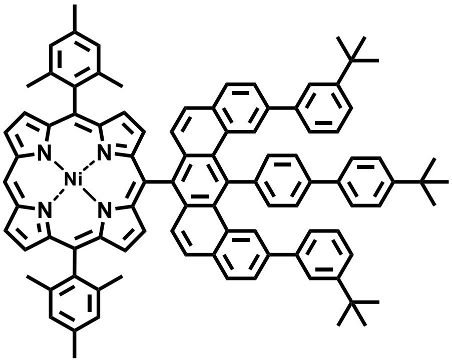

Figure S1. Chemical structure of NGP-1 precursor.

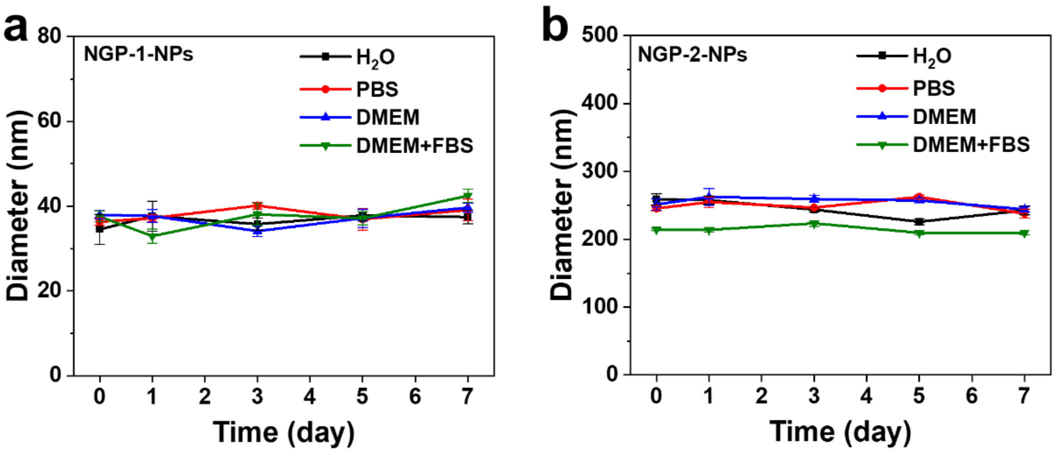

Figure S2. Size distributions of a) NGP-1-NPs and b) NGP-2-NPs by DLS analysis during one-week storage in various aqueous media. The concentration of NGP-1-NPs and NGP-2-NPs was 10 μg mL^{-1}, based on the amount of NGP-1 and NGP-2, respectively, without DSPE-PEG2000. Data shown are presented as mean ± standard deviation (n = 3).

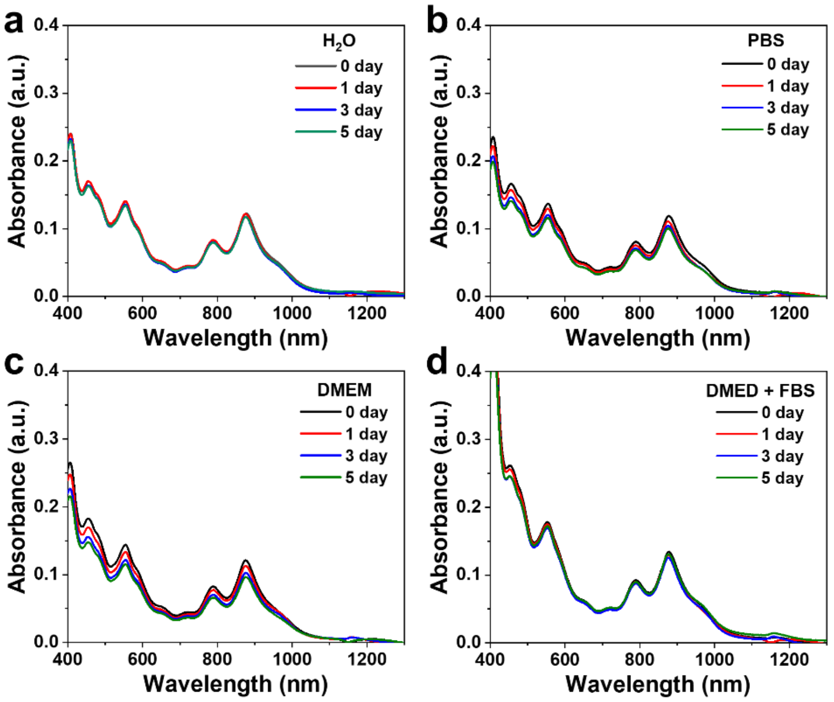

Figure S3. Absorption spectra of NGP-1-NPs (10 μg mL^{-1}, based on the amount of NGP-1 only) during five-day storage in various aqueous media.

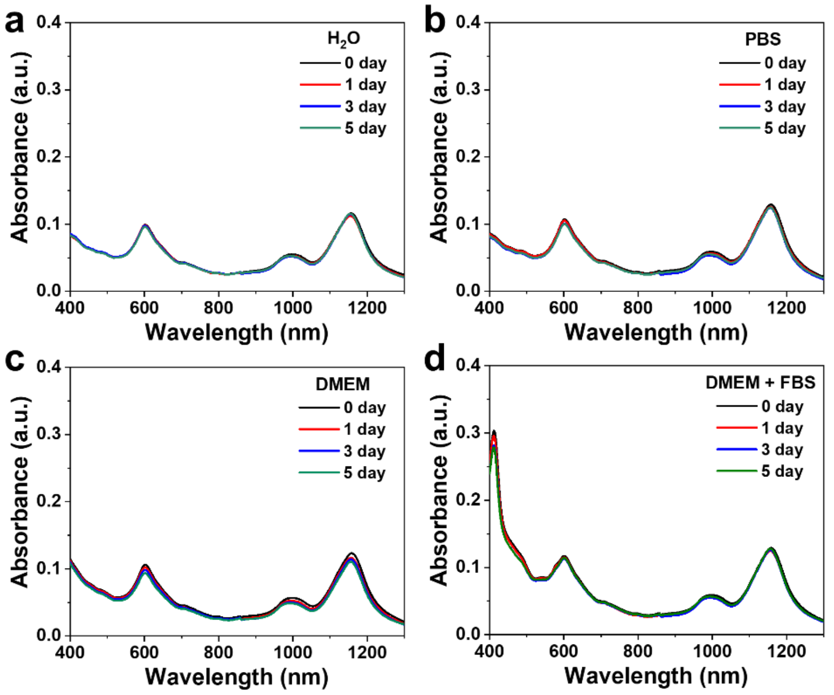

Figure S4. Absorption spectra of NGP-2-NPs (10 μg mL^{-1}, based on the amount of NGP-1 only) during five-day storage in various aqueous media.

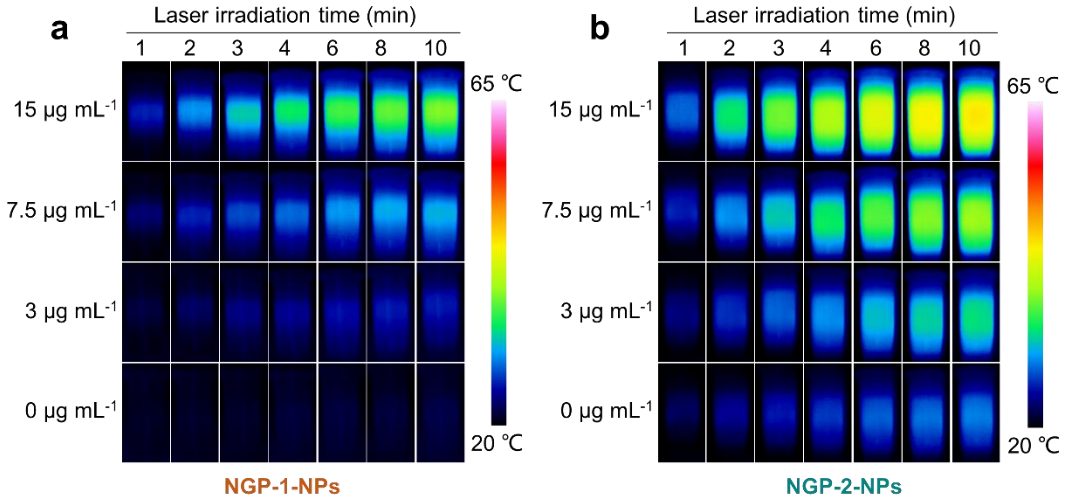

Figure S5. Infrared images of a) NGP-1-NPs and b) NGP-2-NPs dispersions in DI water with various concentration under 808 nm (1 W cm^{-2}) or 1064 nm (1 W cm^{-2}) laser irradiation, respectively.

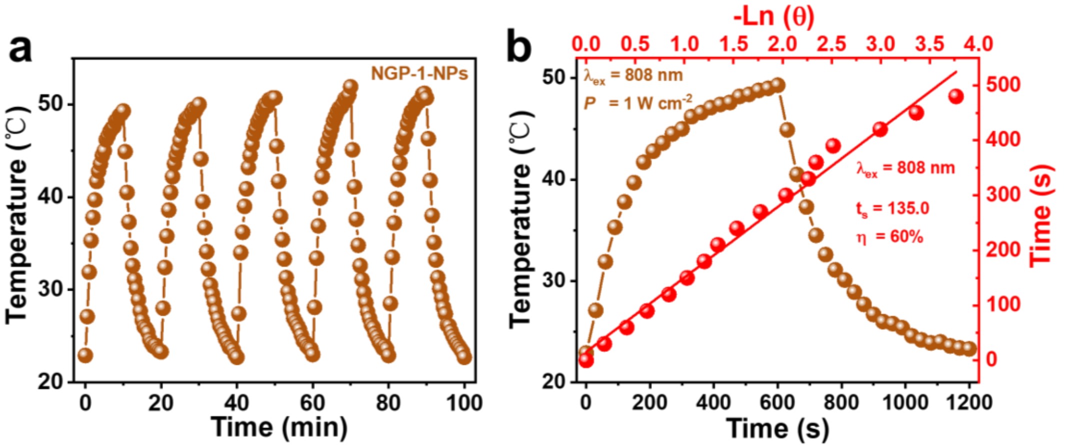

Figure S6. a) Photothermal stability of NGP-1-NPs under 808 nm laser irradiation (1 W cm^{-2}) for five on/off cycles. b) Photothermal performance of NGP-1-NPs by cooling to room temperature with linear analysis. The concentration of NGP-1-NPs was 30 μg mL^{-1}, based on the amount of NGP-1.

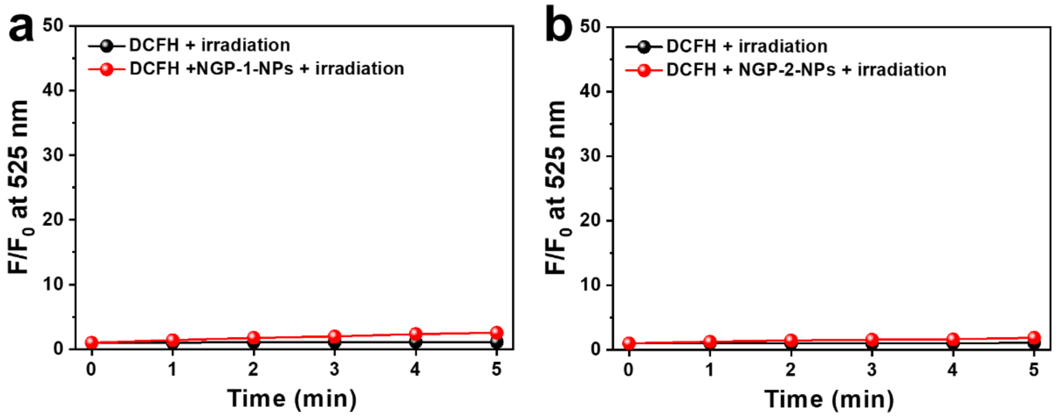

Figure S7. Comparison of the fluorescence rise rate of DCFH solution with or without a) NGP-1-NPs or b) NGP-2-NPs under 808 nm (1 W cm^{-2}) or 1064 nm (1 W cm^{-2}) laser irradiation, respectively.

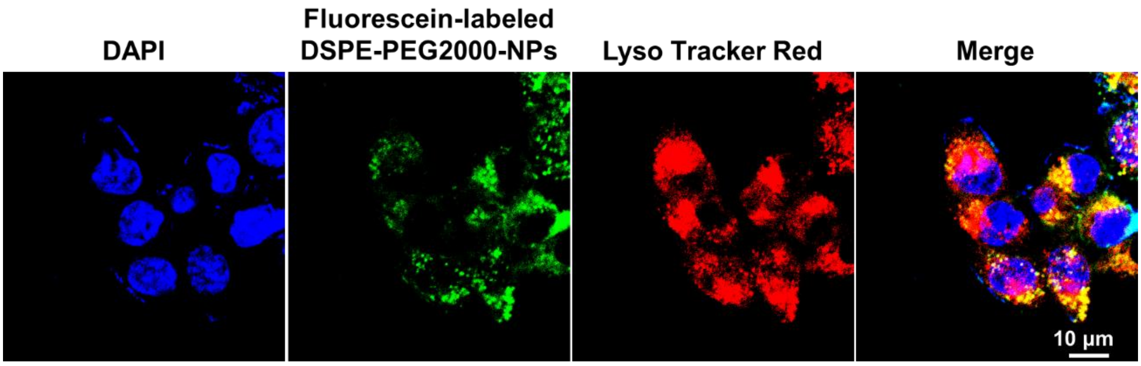

Figure S8. Co-localization of fluorescein-labeled DSPE-PEG2000-NPs with DAPI and LysoTracker after incubating with 4T1 cancer cells for 4 h. Scale bar: 10 μm.

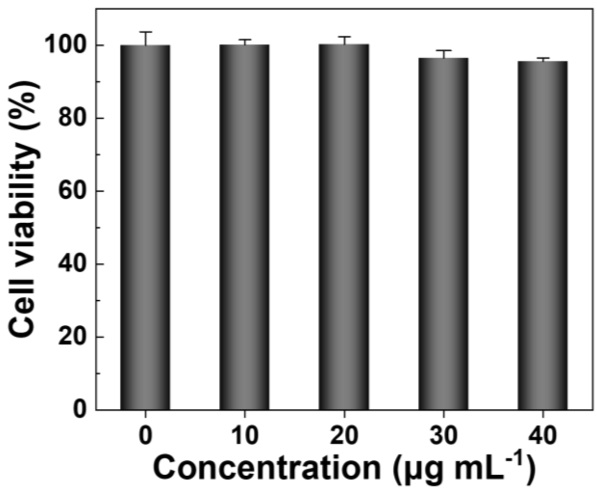

Figure S9. Cell viability of L929 cells treated with various concentrations of NGP-2-NPs in dark for 24 h. Data shown are presented as mean ± standard deviation (*n* = 3).

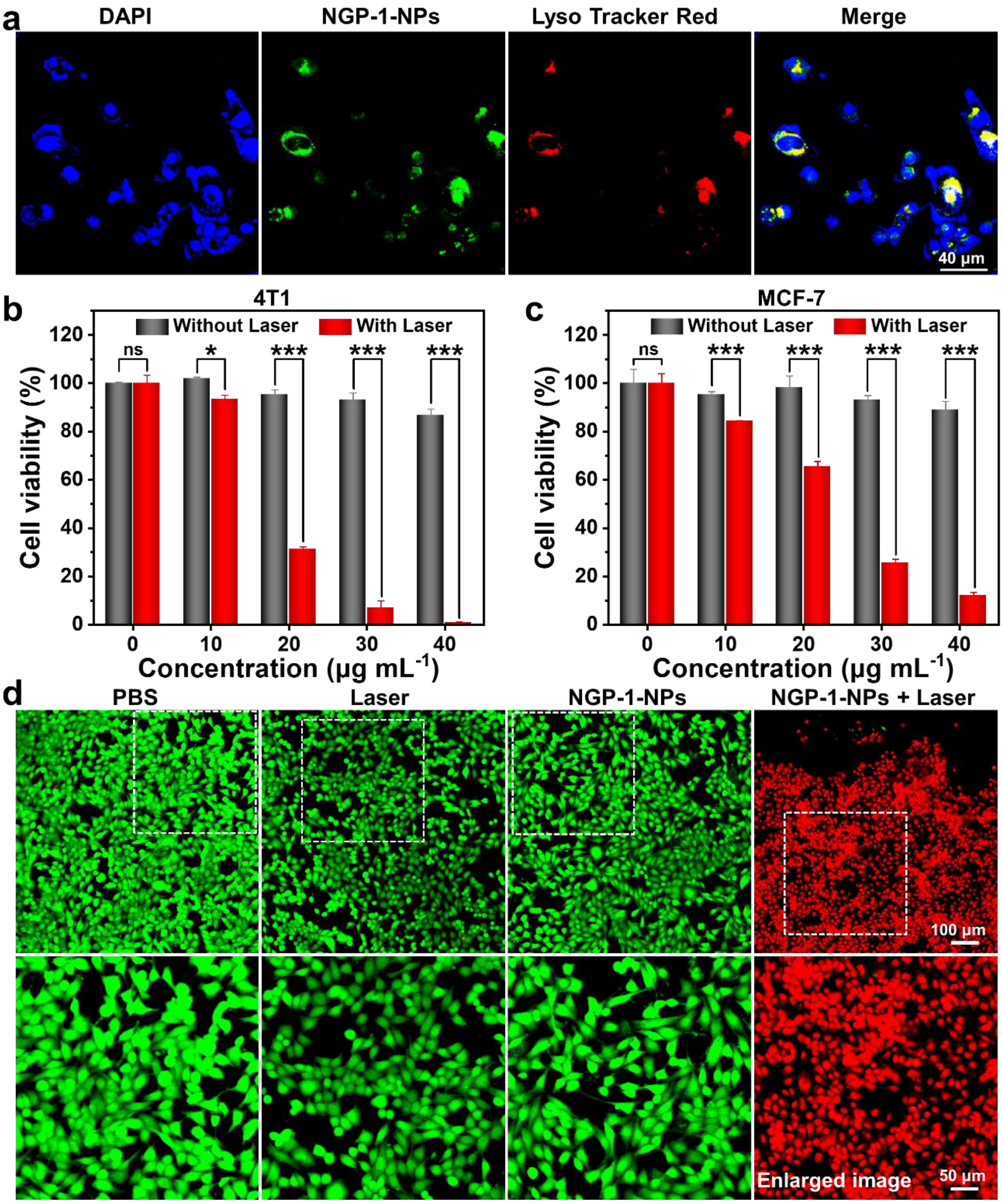

Figure S10. a) Co-localization of NGP-1-NPs with DAPI, and LysoTracker after incubating with 4T1 cancer cells for 4 h. Scale bar: 40 μm. Cell viability of b) 4T1 cells c) and MCF-7 cells treated with various concentrations of NGP-1-NPs with or without 808 nm laser irradiation (1 W cm^{-2}) for 10 min. d) Live/dead images of 4T1 cells costained with AM (green fluorescence for live cells) and PI (red fluorescence for dead cells) after incubation with PBS or NGP-1-NPs with or without 808 nm laser irradiation (1 W cm^{-2}) for 10 min. Scale bar: 100 μm. The corresponding enlarged images for the white box region were also shown, Scale bar: 50 μm. Data shown in panels b and c are presented as mean ± standard deviation (n = 3). P-values are calculated by using one-way ANOVA with Tukey test, ns: not significant, $*P < 0.05$, $**P < 0.01$, $***P < 0.001$.

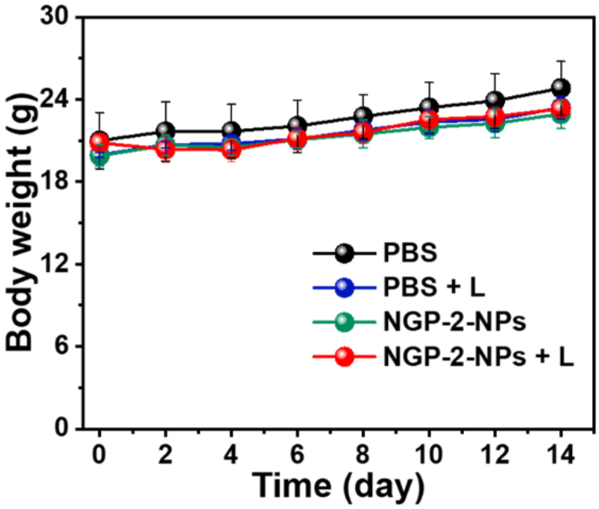

Figure S11. Body weight of mice in different treatment groups during the therapy period. Data are presented as mean ± standard deviation (n = 5).

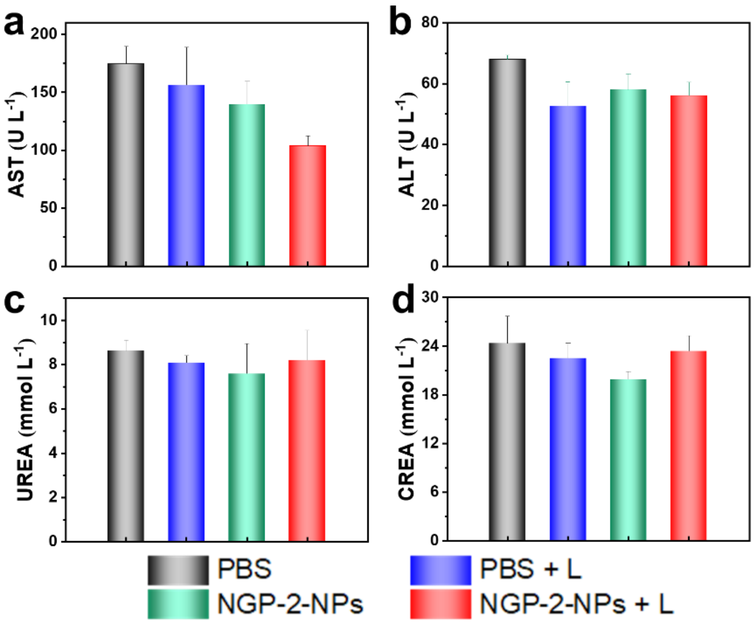

Figure S12. Blood biochemistry tests of different treatment groups. Data are presented as mean ± standard deviation (n = 3).

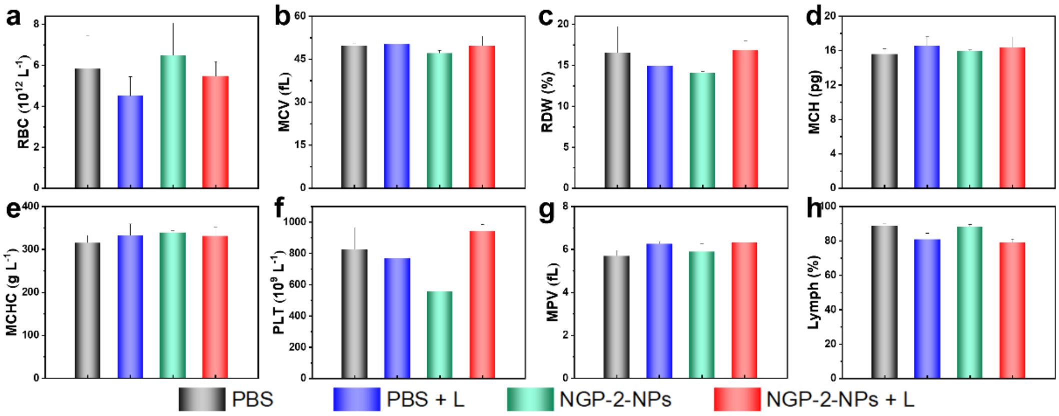

Figure S13. Complete blood panel analysis of different treatment groups. Data are presented as mean ± standard deviation (n = 3).

References

[1] Q. Chen, L. Brambilla, L. Daukiya, K.S. Mali, S. De Feyter, M. Tommasini, K. Müllen, A. Narita, *Angew. Chem. Int. Ed.* **2018**, *57*, 11233.
[2] H. Zhao, X.S. Xu, L. Zhou, Y.B. Hu, Y.M. Huang, A. Narita, *Small* **2022**, *18*, 2105365.
[3] D.K. Roper, W. Ahn, M. Hoepfner, *J. Phys. Chem. C* **2007**, *111*, 3636.
[4] S.L. Li, Q.Y. Deng, X. Li, Y.W. Huang, X.Z. Li, F. Liu, H.J. Wang, W.X. Qing, Z.H. Liu, C.S. Lee, *Biomaterials* **2019**, *216*, 119252.
